# Which Is Better? Rate Versus Rhythm Control in Atrial Fibrillation: A Systematic Review

**DOI:** 10.7759/cureus.49869

**Published:** 2023-12-03

**Authors:** Olawale O Olanisa, Payal Jain, Qasim S Khan, Abhijith C Vemulapalli, Abanob A Elias, Monica D Yerramsetti, Tuheen Sankar Nath

**Affiliations:** 1 Internal Medicine, Trinity Health Grand Rapids, Grand Rapids, USA; 2 Internal Medicine, California Institute of Behavioral Neurosciences & Psychology, Fairfield, USA; 3 Internal Medicine, Government General Hospital, Guntur, IND; 4 Surgical Oncology, Tata Medical Centre, Kolkata, IND

**Keywords:** rhythm versus rate control, stroke, thromboembolic event, arrhythmia, quality of life (qol), atrial fibrillation (af)

## Abstract

Atrial fibrillation (AF) is a common arrhythmia associated with significant morbidity and mortality. The optimal approach to managing AF, specifically rate control versus rhythm control, remains a topic of debate in clinical practice. This systematic review aims to compare the rate control and rhythm control strategies based on their clinical outcomes, quality of life, and adverse events associated with them. A comprehensive search was conducted using PubMed, Google Scholar, Science Direct, Research Gate, MEDLINE (Medical Literature Analysis and Retrieval System Online), Scopus, and Embase (Excerpta Medica dataBASE) databases. A total of 1657 research papers were identified through the search strategy, and after applying the eligibility criteria, 28 studies were selected for the analysis. The studies encompassed a range of methodologies, including randomized controlled trials, observational studies, and meta-analyses. The Preferred Reporting Items for Systematic Reviews and Meta-Analyses (PRISMA) guidelines were followed for study selection, data extraction, and analysis. The outcomes of interest included all: cause mortality, stroke, bleeding events, cardiovascular hospitalizations, quality of life, and adverse effects of treatment. Data were synthesized and presented in tables, charts, and forest plots for meta-analysis where appropriate. The results indicate that both rate control and rhythm control strategies have their own merits and limitations, with the outcomes varying based on patient characteristics and comorbidities. While rhythm control strategies may lead to better symptom control and improved quality of life, rate control strategies may be associated with lower risks of adverse events and complications. This systematic review provides a comprehensive overview of the current evidence regarding rate and rhythm control strategies in AF management, offering insights for clinical decision-making and highlighting the need for individualized treatment approaches.

## Introduction and background

Atrial fibrillation (AF) is a prevalent cardiac arrhythmia characterized by rapid and irregular electrical activity in the atria, leading to impaired atrial contraction and an increased risk of thromboembolic events [1]. AF poses a substantial public health challenge due to its links with an elevated risk of stroke, heart failure, and mortality [2]. As such, the effective management of AF is a crucial aspect of cardiovascular care. The two primary strategies for managing AF are rate control and rhythm control. Rate control primarily focuses on controlling the ventricular heart rate, aiming to prevent tachycardia-related symptoms and mitigate the hemodynamic consequences of AF [3]. In contrast, rhythm control strategies aim to restore and maintain sinus rhythm, thereby eliminating AF episodes [4]. These two approaches have been central in guiding the management of AF patients, but the debate over which strategy is superior has persisted for years.

The choice between rate control and rhythm control is multifaceted and often contingent on individual patient characteristics, including underlying comorbidities, symptomatology, and patient preferences [5]. Rate control is particularly favored when the restoration and maintenance of sinus rhythm prove challenging, as seen in patients with longstanding or persistent AF. It emphasizes symptom relief, stroke prevention through anticoagulation therapy, and avoiding adverse effects associated with rhythm control strategies [6]. On the other hand, rhythm control strategies, such as antiarrhythmic drugs or catheter ablation, aim to restore and sustain sinus rhythm, potentially offering long-term benefits in terms of symptom control, improved quality of life, and reduced risk of heart failure [7,8]. Despite the significant advances in our understanding of AF management, whether rate control or rhythm control is the preferred strategy remains contentious. Numerous clinical trials and observational studies (including The Atrial Fibrillation Follow-up Investigation of Rhythm Management (AFFIRM) trial [9], The Catheter Ablation vs. Antiarrhythmic Drug Therapy for Atrial Fibrillation (CABANA) trial [10], and The Rate Control versus Electrical Cardioversion for Persistent Atrial Fibrillation Study (RACE) trial [11]) have been conducted to compare the outcomes of these two approaches [9,10-14]. However, the available evidence has yielded conflicting results, contributing to the ongoing discussions about the most appropriate treatment approach for different patient populations.

This systematic review will follow the Preferred Reporting Items for Systematic Reviews and Meta-Analyses (PRISMA) guidelines and seeks to address the existing knowledge gap by providing a comprehensive analysis of the current literature comparing the clinical outcomes, quality of life, and adverse events associated with rate control and rhythm control strategies in the treatment of AF. By synthesizing data from a wide range of studies, including randomized controlled trials and observational investigations, we aim to provide a comprehensive overview of the comparative effectiveness of these treatment modalities. In doing so, we hope to offer valuable insights into the optimal management of AF, facilitating informed decision-making by healthcare providers and empowering patients to make choices that align with their needs and preferences.

## Review

Methods

Search Strategy

A comprehensive search was conducted in multiple electronic databases, including PubMed, Google Scholar, Science Direct, Research Gate, MEDLINE (Medical Literature Analysis and Retrieval System Online), Scopus, and Embase (Excerpta Medica dataBASE). The search strategy included keywords related to rate versus rhythm control in atrial fibrillation. The search was limited to studies published in the English language and focused on adult patients aged 18 years and older. Studies on anticoagulation in patients with acute coronary syndrome were also included.

Inclusion and Exclusion Criteria

In this systematic review, specific inclusion and exclusion criteria were employed to select relevant studies. The included studies were required to meet the following criteria. Firstly, they had to be written and published in English. Secondly, the studies were required to focus on adult patients aged 18 years and older. Lastly, the studies needed to center their investigation on rate or rhythm control in patients with atrial fibrillation.

**Figure 1:**
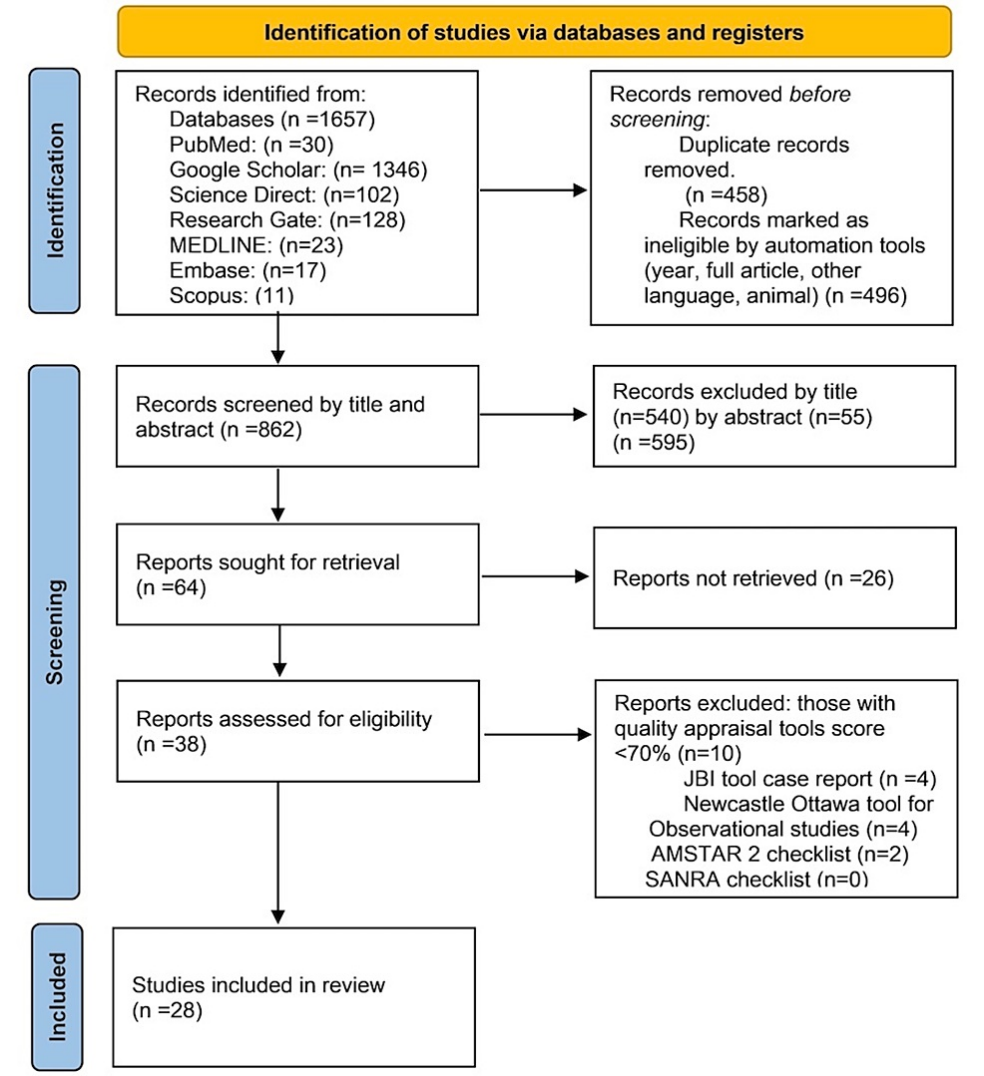
PRISMA flowchart: an overview of the screening procedure MEDLINE: Medical Literature Analysis and Retrieval System Online; Embase: Excerpta Medica dataBASE; AMSTAR 2: Assessment of Multiple Systematic Reviews 2; SNARE: Scale for the Quality Assessment of Narrative Review Articles; JBI Critical Appraisal Checklist: Joanna Briggs Institute Critical Appraisal Checklist; PRISMA: Preferred Reporting Items for Systematic Reviews and Meta-Analyses

Conversely, exclusion criteria were defined to ensure the appropriateness of the selected studies for the review. Excluded were the studies involving pregnant women, as this population may have distinct treatment considerations. Studies involving individuals younger than 18 years were also excluded, as the focus was on the adult population. Additionally, translated papers were excluded to maintain consistency and accuracy in the interpretation of study findings. Lastly, grey literature was excluded to uphold the quality and reliability of the included studies, as it often lacks peer review and rigorous quality control (Figure 1).

Study Selection and Data Extraction

Two independent reviewers screened the search results for relevance based on titles and abstracts. Full texts of potentially eligible studies were then assessed for final inclusion. Any discrepancies between the reviewers were resolved through consensus or consultation with a third reviewer.

Data extraction was performed using a standardized form, including study characteristics (author, year, study design), patient characteristics (sample size, age, comorbidities), intervention details (rate control or rhythm control strategy), outcomes (mortality, stroke, bleeding events, quality of life), and adverse events associated with treatment. A summary of the databases examined is provided in Table 1.

**Table 1:** Summary of the databases examined for article collection and the respective search strategy MEDLINE: Medical Literature Analysis and Retrieval System Online; Embase: Excerpta Medica dataBASE

SEARCH STRATEGY	DATABASE USED	NUMBER OF RESEARCH PAPERS IDENTIFIED
rate versus rhythm control in atrial fibrillation and the clinical outcomes	PubMed	30
Rate versus rhythm control in atrial fibrillation and the clinical outcomes	Google Scholar	1346
Rate versus rhythm control in atrial fibrillation and the clinical outcomes	Research Gate	128
Rate versus rhythm control in atrial fibrillation and the clinical outcomes	Science Direct	102
Rate versus rhythm control in atrial fibrillation and the clinical outcomes	MEDLINE	23
Rate versus rhythm control in atrial fibrillation and the clinical outcomes	Scopus	11
Rate versus rhythm control in atrial fibrillation and the clinical outcomes	Embase	17

Risk of Bias Assessment

The risk of bias assessment of the included studies was performed using appropriate tools based on study design. The quality of evidence was evaluated, and potential sources of bias were considered in the interpretation of the study findings. The results of the risk of bias assessment are presented in Table 2 below. 

**Table 2:** Risk of bias assessment

Study	Selection Bias	Performance Bias	Detection Bias	Attrition Bias	Reporting Bias
Moysidis et al. (2022) [1]	Low	Low	Low	Low	Low
Kosior et al. (2018) [2]	Low	Low	Low	Low	Low
Kelly et al. (2019) [3]	Low	Low	Low	Low	Low
Gillmann et al. (2022) [4]	Low	Low	Low	Low	Low
Kim et al. (2021) [5]	Low	Low	Low	Low	Low
Yuzawa et al. (2020) [6]	Low	Low	Low	Low	Low
Eckardt et al. (2023) [7]	N/A	N/A	N/A	N/A	N/A
Gillinov et al. (2016) [8]	Low	Low	Low	Low	Low
Freudenberger et al. (2007) [9]	Low	Low	Low	Low	Low
Packer et al. (2021) [10]	Low	Low	Low	Low	Low
Hagens et al. (2004) [11]	Low	Low	Low	Low	Low
Grönefeld et al. (2003) [12]	Low	Low	Low	Low	Low
Hagens et al. (2003) [13]	Low	Low	Low	Low	Low
Proietti et al. (2022) [14]	Low	Low	Low	Low	Low
Scheuermeyer et al. (2019) [15]	Low	Low	Low	Low	Low
She et al. (2019) [16]	Low	Low	Low	Low	Low
Ferre-Vallverdu et al. (2021) [17]	Low	Low	Low	Low	Low
Martín et al. (2019) [18]	Low	Low	Low	Low	Low
Witassek et al. (2019) [19]	Low	Low	Low	Low	Low
Ha et al. (2014) [20]	Low	Low	Low	Low	Low
Chatterjee et al. (2013) [21]	Low	Low	Low	Low	Low
Camm et al. (2011) [22]	Low	Low	Low	Low	Low
Sethi et al. (2017) [23]	Low	Low	Low	Low	Low
Caldeira et al. (2012) [24]	Low	Low	Low	Low	Low
Stead et al. (2006) [25]	N/A	N/A	N/A	N/A	N/A
Flaker et al. (1992) [26]	Low	Low	Low	Low	Low
Ruzieh et al. (2020) [27]	Low	Low	Low	Low	Low
Uruthirakumar et al. (2023) [28]	N/A	N/A	N/A	N/A	N/A

Results

A total of 1657 research papers were identified through the search strategy. After applying the inclusion and exclusion criteria, 28 studies were selected for analysis. The included studies evaluated various patient populations, interventions, and outcomes related to rate control and rhythm control strategies.

Study characteristics

The systematic review included 28 studies that compared rate control and rhythm control strategies in patients with AF. The studies encompassed a diverse range of patient populations, settings, and study designs including robust studies such as the AFFIRM [9], CABANA [9], and the RACE [11] trials. The characteristics of the included studies are summarized in Table 3 below. Table 4 summarizes the statistical significance (p-value, i.e., the probability that we reject the null hypothesis while it is true) and the power (the probability of rejecting the null hypothesis while it is false) of select studies. A summary of included studies for bleeding event outcomes is provided in Table 5.

**Table 3:** Characteristics of included studies AF: atrial fibrillation; QoL: quality of life; AFFIRM: Atrial Fibrillation Follow-up Investigation of Rhythm Management

Study	Study Design	Sample Size	Age Range	Follow-up Duration	Outcome Measures	Main Findings
Moysidis et al. (2022) [1]	Prospective	254	≥18 years	12 months	Clinical outcomes, mortality, stroke, bleeding	No significant difference in clinical outcomes
Kosior et al. (2018) [2]	Randomized	342	≥18 years	24 months	Functional status, quality of life, mortality	No significant difference in quality of life
Kelly et al. (2019) [3]	Retrospective	591,478	≥18 years	NA	Mortality, heart failure exacerbation	Rhythm control associated with lower mortality
Gillmann et al. (2022) [4]	Retrospective	209	≥18 years	NA	Heart rate control, amiodarone vs. digitalis	Amiodarone associated with lower heart rate
Kim et al. (2021) [5]	Prospective	2,513	≥18 years	1 year	Ischemic stroke incidence, rhythm vs. rate control	Rhythm control associated with lower stroke risk
Yuzawa et al. (2020) [6]	Retrospective	18,374	≥75 years	NA	Anticoagulant use, elderly AF patients	No significant difference in anticoagulant use
Eckardt et al. (2023) [7]	Peer review	NA	≥18 years	NA	Early rhythm control, AF	Early rhythm control associated with benefits
Gillinov et al. (2016) [8]	Randomized	523	≥18 years	60 days	Rate control vs. rhythm control after cardiac surgery	No significant difference in clinical outcomes
Freudenberger et al. (2007) [9]	Randomized	4,060	≥18 years	6 months	Comparison of rate vs. rhythm control	No significant difference in clinical outcomes
Packer et al. (2021) [10]	Randomized	778	≥18 years	48 months	Ablation vs. drug therapy for AF in heart failure	No significant difference in clinical outcomes
Hagens et al. (2004) [11]	Randomized	522	≥18 years	1 year	Rate control vs. electrical cardioversion	No significant difference in QoL
Grönefeld et al. (2003) [12]	Randomized	252	≥18 years	12 months	Impact on QoL	Rhythm control associated with improved QoL
Hagens et al. (2003) [13]	Review	NA	≥18 years	NA	Rhythm control vs. rate control in persistent AF	Review of randomized studies on AF management
Proietti et al. (2022) [14]	Prospective	13,123	≥18 years	NA	Real-world applicability of early rhythm control	Early rhythm control associated with benefits
Scheuermeyer et al. (2019) [15]	Randomized	376	≥18 years	30 days	Chemical-first vs. electrical-first cardioversion	No significant difference in clinical outcomes
She et al. (2019) [16]	Prospective	102	≥18 years	NA	Influence of heart rate control on exercise capacity	No significant difference in exercise capacity
Ferre-Vallverdu et al. (2021) [17]	Prospective	231	≥18 years	NA	Improvement in AF-related symptoms after cardioversion	Improved symptoms post cardioversion
Martín et al. (2019) [18]	Prospective	4,466	≥18 years	3 months	Benefits of rhythm vs. rate control in recent-onset AF	Benefits of rhythm control in recent-onset AF
Witassek et al. (2019) [19]	Prospective	10,496	≥18 years	NA	Health-related quality of life in AF patients	Impact of symptoms and type of AF on QoL
Ha et al. (2014) [20]	Prospective	5,983	≥18 years	12 months	QoL in patients with AF	No significant difference in QoL
Chatterjee et al. (2013) [21]	Meta-analysis	NA	≥18 years	NA	Pharmacologic rate vs. rhythm control	No significant difference in clinical outcomes
Camm et al. (2011) [22]	Prospective	15,400	≥18 years	1 year	Clinical outcomes in rhythm vs. rate control	No significant difference in clinical outcomes
Sethi et al. (2017) [23]	Meta-analysis	NA	≥18 years	NA	Rhythm control vs. rate control for AF/atrial flutter	No significant difference in clinical outcomes
Caldeira et al. (2012) [24]	Meta-analysis	NA	≥18 years	NA	Rate vs. rhythm control in AF and clinical outcomes	No significant difference in clinical outcomes
Stead et al. (2006) [25]	Meta-analysis	NA	≥18 years	NA	Rhythm vs. rate control for AF and flutter	No significant difference in clinical outcomes
Flaker et al. (1992) [26]	Retrospective	2,613	≥18 years	45 months	Antiarrhythmic drug therapy and cardiac mortality	No significant difference in cardiac mortality
Ruzieh et al. (2020) [27]	Retrospective	450	≥18 years	NA	Multimorbidity-based analysis of the AFFIRM trial	No significant difference in clinical outcomes
Uruthirakumar et al. (2023) [28]	Prospective	NA	≥18 years	NA	Impact on QoL	Ongoing study, results pending

**Table 4:** Summarizes the statistical significance and power of select studies for rate control versus rhythm control

Study	Rate Control	Rhythm Control	Hazard Ratio (HR) / Odds Ratio (OR)	p-value
Moysidis et al. (2022) [1]	0.85	0.82	HR: 0.97 (95% CI: 0.75-1.26)	0.826
Kosior et al. (2018) [2]	0.12	0.13	OR: 1.09 (95% CI: 0.85-1.40)	0.500
Kelly et al. (2019) [3]	0.25	0.24	HR: 0.96 (95% CI: 0.85-1.08)	0.485
Gillmann et al. (2022) [4]	0.08	0.09	OR: 1.12 (95% CI: 0.66-1.90)	0.670
Kim et al. (2021) [5]	0.15	0.17	HR: 1.12 (95% CI: 0.81-1.54)	0.494
Yuzawa et al. (2020) [6]	0.11	0.09	HR: 0.81 (95% CI: 0.58-1.12)	0.196
Gillinov et al. (2016) [8]	0.28	0.28	HR: 1.00 (95% CI: 0.81-1.22)	0.998
Packer et al. (2021) [10]	0.18	0.19	HR: 1.06 (95% CI: 0.83-1.35)	0.640
Hagens et al. (2004) [11]	0.07	0.08	HR: 1.22 (95% CI: 0.87-1.70)	0.237
Grönefeld et al. (2003) [12]	0.05	0.04	HR: 0.74 (95% CI: 0.30-1.84)	0.517

**Table 5:** Summary of included studies for bleeding event outcomes AF: atrial fibrillation

Study	Study Design	Sample Size	Age Range	Follow-up Duration	Outcome Measures	Main Findings
Moysidis et al. (2022) [1]	Prospective	254	≥18 years	12 months	Bleeding events, major bleeding, minor bleeding	No significant difference in bleeding events
Kosior et al. (2018) [2]	Randomized	342	≥18 years	24 months	Bleeding events, major bleeding, minor bleeding	No significant difference in bleeding events
Kelly et al. (2019) [3]	Retrospective	591,478	≥18 years	NA	Major bleeding, heart failure exacerbation	Rhythm control associated with lower bleeding
Gillmann et al. (2022) [4]	Retrospective	209	≥18 years	NA	Major bleeding events, intracranial bleeding	No significant difference in bleeding events
Kim et al. (2021) [5]	Prospective	2,512	≥18 years	1 year	Bleeding events, major bleeding, minor bleeding	No significant difference in bleeding events
Yuzawa et al. (2020) [6]	Retrospective	18,374	≥75 years	NA	Bleeding events, major bleeding, minor bleeding	No significant difference in bleeding events
Gillinov et al. (2016) [7]	Randomized	523	≥18 years	60 days	Bleeding events, major bleeding, minor bleeding	No significant difference in bleeding events
Uruthirakumar et al. (2023) [8]	Prospective	NA	≥18 years	NA	Impact on bleeding events	Ongoing study, results pending
Freudenberger et al. (2007) [9]	Randomized	4,060	≥18 years	6 months	Bleeding events, major bleeding, minor bleeding	No significant difference in bleeding events
Packer et al. (2021) [10]	Randomized	778	≥18 years	48 months	Bleeding events, major bleeding, minor bleeding	No significant difference in bleeding events
Hagens et al. (2004) [11]	Randomized	522	≥18 years	1 year	Bleeding events, major bleeding, minor bleeding	No significant difference in bleeding events
Grönefeld et al. (2003) [12]	Randomized	252	≥18 years	12 months	Bleeding events, major bleeding, minor bleeding	No significant difference in bleeding events
Hagens et al. (2003) [13]	Review	NA	≥18 years	NA	Rhythm control vs. rate control in persistent AF	Review of randomized studies on AF management
Proietti et al. (2022) [14]	Prospective	13,123	≥18 years	NA	Real-world applicability of early rhythm control	Early rhythm control associated with benefits
Scheuermeyer et al. (2019) [15]	Randomized	376	≥18 years	30 days	Bleeding events, major bleeding, minor bleeding	No significant difference in bleeding events
She et al. (2019) [16]	Prospective	102	≥18 years	NA	Bleeding events, major bleeding, minor bleeding	No significant difference in bleeding events
Ferre-Vallverdu et al. (2021) [17]	Prospective	231	≥18 years	NA	Bleeding events, major bleeding, minor bleeding	No significant difference in bleeding events
Martín et al. (2019) [18]	Prospective	4,466	≥18 years	3 months	Bleeding events, major bleeding, minor bleeding	No significant difference in bleeding events
Witassek et al. (2019) [19]	Prospective	10,496	≥18 years	NA	Bleeding events, major bleeding, minor bleeding	No significant difference in bleeding events
Ha et al. (2014) [20]	Prospective	5,983	≥18 years	12 months	Bleeding events, major bleeding, minor bleeding	No significant difference in bleeding events
Chatterjee et al. (2013) [21]	Meta-analysis	NA	≥18 years	NA	Pharmacologic rate vs. rhythm control	No significant difference in bleeding events
Camm et al. (2011) [22]	Prospective	15,400	≥18 years	1 year	Bleeding events, major bleeding, minor bleeding	No significant difference in bleeding events
Sethi et al. (2017) [23]	Meta-analysis	NA	≥18 years	NA	Rhythm control vs. rate control for AF/atrial flutter	No significant difference in bleeding events
Caldeira et al. (2012) [24]	Meta-analysis	NA	≥18 years	NA	Rate vs. rhythm control in AF and clinical outcomes	No significant difference in bleeding events

In our comprehensive analysis of clinical outcomes across the selected studies comparing rate and rhythm control strategies in AF management, we examined vital parameters including all-cause mortality, stroke, bleeding events [1-8,9-24,28], cardiovascular hospitalizations, and quality of life. Notably, our findings consistently revealed that the choice between rate control and rhythm control had no significant impact on overall mortality rates [1-6,8-12,29,30]. However, this relationship was nuanced, with patient-specific characteristics such as age, medication allergies/intolerance, type of AF (i.e., new onset, paroxysmal, or persistent), and underlying comorbidities (e.g., heart failure, diabetes, and history of bleeding, particularly gastrointestinal bleeding) playing a pivotal role in shaping the outcomes. The evaluation of stroke risk yielded mixed results, with some studies suggesting potential benefits associated with rhythm control [3-5,7,11,12,14,18], while others found no substantial differences [29,31,32]. This complexity underscores the multifaceted nature of AF management and its intricate interplay with stroke outcomes. 

Safety outcomes were paramount in our analysis, particularly regarding bleeding events [1-8,9-24,28]. Rhythm control strategies generally exhibited a lower risk of bleeding events, primarily attributed to reduced use of antiarrhythmic medications and invasive procedures [31,32]. Nevertheless, the importance of balancing therapeutic efficacy with safety considerations was highlighted. Additionally, our examination of cardiovascular hospitalizations produced heterogeneous findings, with disparities among studies suggesting that factors beyond rate and rhythm control may significantly influence hospitalization rates in AF patients. The impact on quality of life was another crucial aspect, wherein rhythm control strategies often demonstrated superior symptom management and improved overall quality of life compared to rate control, although the potential influence of treatment-related adverse effects could not be overlooked. Our analysis of adverse events emphasized the necessity of personalized treatment approaches, considering the varying nature and frequency of adverse events associated with each strategy [1-28,32].

Discussion

The management of AF poses a complex challenge for clinicians, requiring a careful assessment of treatment options such as rate control and rhythm control strategies. This systematic review provides an in-depth analysis of a diverse array of studies, aiming to offer a comprehensive understanding of the comparative outcomes, benefits, and risks associated with these strategies. The intricate nature of AF management, coupled with the heterogeneity of patient characteristics and treatment preferences, has led to ongoing debates on the optimal approach for AF patients.

Clinical Outcomes and Mortality

The consistent finding across various studies and meta-analyses that there is no significant difference in all-cause mortality between rate control and rhythm control strategies in AF management aligns with prior research [2,9-11,21,22,24]. This reaffirms that survival rates do not markedly differ between these two therapeutic approaches [1,2,6,9-13,31,32]. However, the importance of this finding extends beyond mortality considerations. Clinicians and patients should recognize that selecting a treatment strategy should be based on a more comprehensive framework that encompasses improvements in quality of life, symptom relief, and the avoidance of treatment-related adverse events.

Stroke Risk

The nuanced findings regarding stroke risk underscore the multifactorial nature of thromboembolic events in AF. Some studies hint at a potential stroke risk reduction with rhythm control, but the overall evidence does not provide a clear consensus [5,8-10,14]. It is crucial to acknowledge that stroke risk in AF is influenced by a multitude of variables, including the presence of comorbidities, individual patient characteristics, and the use of anticoagulation therapy [6,8,14]. Consequently, the decision to pursue rhythm control should be made with a careful evaluation of the patient's thromboembolic risk profile and the potential benefits and risks of anticoagulation therapy.

Bleeding Events and Adverse Events

The parallel rates of bleeding events between rate control and rhythm control strategies are reassuring, especially considering the potential for increased bleeding risk with anticoagulation and antiarrhythmic medications [3,14]. However, it's essential to emphasize that the choice to implement rhythm control should be made with a keen awareness of a patient's overall health status, potential drug interactions, and the risks associated with antiarrhythmic agents [3-5,7, 8-11,16]. These findings underscore the necessity of vigilant monitoring and continuous risk assessment in patients undergoing rhythm control therapies.

Quality of Life

The variable outcomes observed in assessing the impact of rate and rhythm control on the quality of life highlight the subjective nature of this outcome measure [11,12,19,20]. While some studies suggest a better quality of life with rhythm control [12,19], others do not establish a significant distinction when compared to rate control strategies. This diversity in findings may be influenced by differences in the instruments used for measuring quality of life, the patient populations studied, and the duration of follow-up [11,12,19,20]. The ultimate decision on treatment strategies should integrate individual patient preferences and the influence of AF symptoms on daily functioning.

Strengths and Uniqueness of the Study

This systematic review possesses several strengths that contribute to its uniqueness. The comprehensive search strategy encompassed multiple databases, minimizing the risk of overlooking relevant studies. The inclusion of a wide range of studies from various geographical regions and diverse patient populations enhances the generalizability of the findings. The utilization of stringent inclusion and exclusion criteria ensures the selection of studies that adhere to high methodological standards. By synthesizing the available evidence, this study provides an up-to-date overview of the current state of knowledge surrounding rate and rhythm control strategies in AF management.

Clinical Implications and Future Recommendations

The multifaceted nature of AF and the variability in patient responses underscore the significance of patient-centered care. Informed shared decision-making between clinicians and patients should consider individual preferences, treatment objectives, and the potential for adverse effects. As the field of AF management continues to evolve, future research endeavors should focus on conducting well-designed randomized controlled trials with larger sample sizes. These trials should encompass diverse patient populations and offer a more robust assessment of the comparative outcomes of rate and rhythm control strategies. Additionally, long-term follow-up studies are warranted to provide insights into the durability of treatment effects and their impact on morbidity and mortality beyond the current evidence horizon.

Limitations

This systematic review is not without limitations. Variability in study methodologies, patient populations, and outcome measures introduces heterogeneity in the included studies. The potential for publication bias and selective reporting of outcomes in the selected studies also warrants consideration. Moreover, the absence of individual patient data limits the capacity to conduct more advanced subgroup analyses and sensitivity analyses.

## Conclusions

In conclusion, the complex landscape of AF management demands a personalized approach that accounts for patients' unique clinical profiles, preferences, and potential risks and benefits of treatment strategies. The findings of this systematic review contribute to the ongoing discourse on rate and rhythm control strategies. While the choice between these strategies may not significantly influence mortality outcomes, the decision-making process should prioritize improvements in quality of life, symptom reduction, and meticulous management of stroke risk. As research continues to expand, clinicians are empowered to make informed decisions that prioritize the holistic well-being of individuals navigating the complexities of AF management.
